# Intrascrotal extratesticular schwannoma

**DOI:** 10.4103/0970-9371.66696

**Published:** 2010-01

**Authors:** Viral Bhanvadia, PM Santwani

**Affiliations:** Department of Pathology, Shree M. P. Shah Medical College, Jamnagar, Gujarat, India

**Keywords:** Fine needle aspiration cytology, schwannoma, scrotum

## Abstract

Primary nerve sheath tumors in an intrascrotal and extratesticular location are extremely rare, with only a few cases reported in the literature. We report a fine needle aspiration cytology (FNAC)-diagnosed and histologically confirmed case of paratesticular schwannoma in a 32-year-old male. The patient had no evidence of neurofibromatosis and there was a pre-operative cytologic suspicion of leiomyoma as well. A palpable left-sided inguinal lymph node showed features of reactive lymphoid hyperplasia. This case is documented to highlight the rarity of paratesticular schwannoma and the role of FNAC in its pre-operative interpretation.

## Introduction

Although documented in the literature, schwannomas of the scrotum and testis are rare. Most of them occur during the 3^rd^ and 4^th^ decades of life, with an equal gender distribution.[1] Cytological features of schwannomas are well documented in the literature.[2–7] A representative cytologic material with the presence of cells exhibiting wavy, comma-shaped nuclei, haphazardly placed in a myxoid matrix as well as nuclear palisades at the border of pink fibrillary material resembling verocay bodies can prompt a confident pre-operative diagnosis of schwannoma.[4] Though cases of paratesticular schwannoma have been reported earlier,[7,8] the diagnosis on the basis of fine needle aspiration cytology (FNAC) to the best of our knowledge, has not been documented. We report one such case.

## Case Report

A 32-year-old male presented with a history of gradually increasing, painless, left-sided scrotal swelling of six months’ duration. No similar swellings were appreciated elsewhere in the body and there was no other significant history or clinical findings. On physical examination, an oblong, firm mass, measuring 4.5 cm × 3.5 cm in size was observed at the root of the scrotum, close to the testis. The patient also had a friction ulcer over the scrotal skin along with an enlarged left-sided inguinal lymph node. A provisional clinical diagnosis of epididymal tumor/lymphoma was considered.

Ultrasonographic examination demonstrated the paratesticular location of the scrotal mass with scant amount of fluid collected within the tunica vaginalis, along with an enlarged left-sided inguinal lymph node.

**Figure 1:**
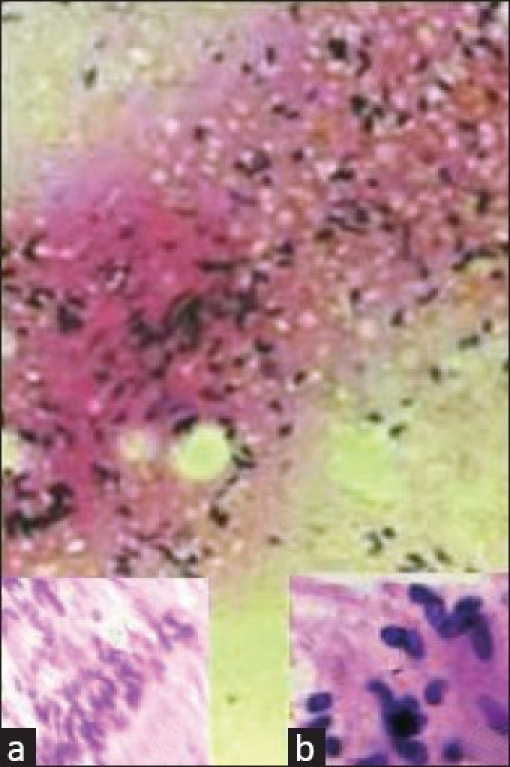
The photomicrograph shows scattered spindle to oval cells amidst a myxomatous background associated with some histiocytes and lymphocytes. Inset 1a shows palisades of cells at the margin of the pinkish material (H and E, ×400). Inset 1b shows the verucay body (H and E, ×400)

FNA of the scrotal mass as well as the lymph node was performed using a 23-gauge needle attached to a 5-mL syringe. Smears were fixed with ether–alcohol (v/v) and stained with hematoxylin and eosin (H and E). Examination of the cytologic smears revealed tightly cohesive tissue fragments with variable cellularity, exhibiting slender spindle cells showing dark, wavy nuclei [Figure 1]. Some clusters showed palisades of nuclei [Figure 1a] at the margin of the pinkish material simulating verocay bodies [Figure 1b]. Also seen were scattered spindle to oval cells amidst the myxomatous background along with some histiocytes and lymphocytes [Figure 1]. The spindle cells showed “fish hook” nuclei, some of which were seen in pairs. Based on these findings, a cytological diagnosis of schwannoma was offered with a differential diagnosis of leiomyoma. Aspirates from the lymph node showed reactive lymphoid hyperplasia.

**Figure 2:**
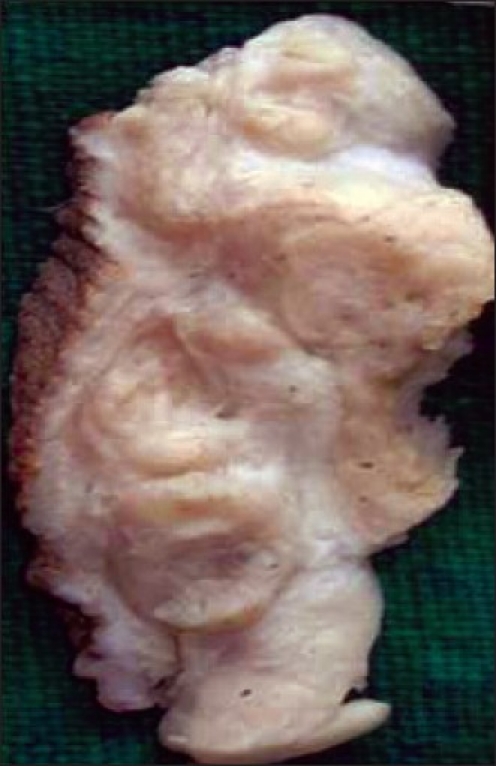
Cut-surface of the mass shows whitish, nodular, round, glistening areas with yellow-brown soft areas in the centre

Surgical exploration of the scrotal sac revealed a firm, nodular, whitish lesion attached to the sac and not involving the testis. Scrotal mass was excised sparing the testis. The specimen submitted for histopathological examination consisted of a partly skin-covered, oblong, firm mass, which measured 4.5 cm × 3.5 cm × 2 cm. The cut-surface was whitish, lobulated and glistening, with central yellow-brown areas [Figure 2]. Imprints taken from the cut-surface of the tumor were pauci-cellular; however, showing appreciable verocay bodies. H and E-stained sections showed classic features of schwannoma with Antony A and Antony B areas.

## Discussion

Schwannomas arise from the cranial, spinal and the peripheral nerves of the body. Several sites of involvement have been documented, with the rare sites reported being face, neck, scalp, hands, tongue, palate and the larynx. Testicular schwannomas are the rarest.[1,7,8] FNAC features have been well documented by various authors.[2–4]

Domanski *et al*.[2] studied 116 cases of schwannomas using FNAC as the pre-operative diagnostic tool. The classical cytomorpholoical features described in schwannomas include spindle, wavy nuclei and verocay bodies.[4,5] Sparsely cellular myxomatous material on smears represent type B areas while cellular areas with nuclear palisades along the fibrillary material correspond to Antony A areas. Presence of small round cells with variable amount of cytoplasm arranged in compact nests or short cords are also described.[2–4,9] The diagnosis of the usual type of schwannoma is easy, but diagnosis of variants like cellular, ancient and plexiform schwannoma is often difficult even on histology. In cases with diagnostic dilemma with other spindle cell tumors, an appropriate panel of immunomarkers along with S-100 protein is of great value.[9]

Clinically, schwannomas tend to be asymptomatic; however, when they enlarge, they result in symptoms related to compression of the surrounding structures.[10] Recurrence of the tumor following excision is uncommon.[10] Similarly, malignant transformation in a schwannoma is extremely rare. In the present case, the clinical suspicion was that of an epididymal tumor/lymphoma, while the radiologic impression was that of a paratesticular tumor. There was no evidence of neurofibromatosis.

FNA in our case provided an accurate pre-operative diagnosis. Although a differential of leiomyoma was also considered, it could easily be excluded by the cytological features, which well reflected the Antony A and B areas of the histologic sections. Imprints taken from the surgical specimen, although sparsely cellular, showed features consistent with schwannoma.

Our case highlights the importance of FNA in the pre-operative tissue diagnosis of unusual paratesticular tumors such as schwannoma.
